# Instrumentational Complexity of Music Genres and Why Simplicity Sells

**DOI:** 10.1371/journal.pone.0115255

**Published:** 2014-12-31

**Authors:** Gamaliel Percino, Peter Klimek, Stefan Thurner

**Affiliations:** 1 Section for Science of Complex Systems, CEMSIIS, Medical University of Vienna, Austria; 2 Santa Fe Institute, Santa Fe, New Mexico, United States of America; 3 IIASA, Laxenburg, Austria; National Scientific and Technical Research Council (CONICET), Argentina

## Abstract

Listening habits are strongly influenced by two opposing aspects, the desire for variety and the demand for uniformity in music. In this work we quantify these two notions in terms of instrumentation and production technologies that are typically involved in crafting popular music. We assign an ‘instrumentational complexity value’ to each music style. Styles of low instrumentational complexity tend to have generic instrumentations that can also be found in many other styles. Styles of high complexity, on the other hand, are characterized by a large variety of instruments that can only be found in a small number of other styles. To model these results we propose a simple stochastic model that explicitly takes the capabilities of artists into account. We find empirical evidence that individual styles show dramatic changes in their instrumentational complexity over the last fifty years. ‘New wave’ or ‘disco’ quickly climbed towards higher complexity in the 70s and fell back to low complexity levels shortly afterwards, whereas styles like ‘folk rock’ remained at constant high instrumentational complexity levels. We show that changes in the instrumentational complexity of a style are related to its number of sales and to the number of artists contributing to that style. As a style attracts a growing number of artists, its instrumentational variety usually increases. At the same time the instrumentational uniformity of a style decreases, i.e. a unique stylistic and increasingly complex expression pattern emerges. In contrast, album sales of a given style typically increase with decreasing instrumentational complexity. This can be interpreted as music becoming increasingly formulaic in terms of instrumentation once commercial or mainstream success sets in.

## Introduction

The composer Arnold Schönberg held that joy or excitement in listening to music originates from the struggle between two opposing impulses, ‘the demand for repetition of pleasant stimuli, and the opposing desire for variety, for change, for a new stimulus.’ [1]. These two driving forces – the demand for repetition or uniformity and the desire for variety – influence not only how we perceive popular music, but also how it is produced. This can be seen e.g. in one of last year's critically most acclaimed albums, Daft Punk's *Random Access Memories*. At the beginning of the production process of the album the duo behind Daft Punk felt that the electronic music genre was in its ‘comfort zone and not moving one inch’ [2]. They attributed this ‘identity crisis’ to the fact that artists in this genre mostly miss the tools to create original sounds and rely too heavily on computers with the same libraries of sounds and preset banks [3]. *Random Access Memories* was finally produced with the help of 27 other featured artists or exceptional session musicians, who were asked to play riffs and individual patterns to give the duo a vast library to select from [4]. The percussionist stated that he used ‘every drum he owns’ on the album; there is also a track composed of over 250 different elements. The record was awarded the ‘Album of the Year 2013’ Grammy and received a Metacritic review of ‘universal acclaim’ for, e.g. ‘breath[ing] life into the safe music that dominates today's charts’ [5]. However, the best-selling album of 2013 in the US was not from Daft Punk, but *The 20/20 Experience* by Justin Timberlake. The producer of this album, Timothy Mosley, contributed 25 Billboard Top 40 singles between 2005–2010, more than any other producer [6]. All these records featured a unique production style consisting of ‘vocal sounds imitating turntable scratching, quick keyboard arabesques, grunts as percussion’ [7]. Asked about his target audience, Mosley said ‘I know where my bread and butter is at. […] I did this research. It's the women who watch Sex and the City’ [8]. These two anecdotes illustrate how Schönberg's two opposing forces, the demand for both uniformity and variety, influence the crafting of popular music. The Daft Punk example suggests that innovation and increased variety is closely linked to the involved musicians' skills and thereby to novel production tools and technologies. The example of Mosley shows how uniformity in stylistic expressions can satisfy listener demands and produce large sales numbers over an extended period of time. There is indeed substantial evidence now that it is the delicate balance between repetitiveness and surprise that shapes our emotional responses to music [9], [10].

The complexity of music is a multi-faceted concept [11]. Aspects of this complexity that are amenable to a quantitative evaluation include acoustics (the dynamic range and the rate of change in dynamic levels of audio tracks), timbre (the source of the sound and the way that this source is excited), as well as complexity measures for the melodic, harmonic, and rhythmic content of music (that are often based on time-frequency analyses) [12]. The so-called ‘optimal complexity hypothesis’ suggests that audiences prefer music of intermediate perceived complexity [13], as has recently been experimentally confirmed for modern jazz piano improvisations [14]. It is worth to note that commercial success or popularity of music (as measured by the numbers of sales or listeners, respectively) is not determined by quality or complexity of music alone [15]. The number of record sales of a given artist is in general also not correlated with the record sales of similar artists [16]. In an ‘artificial music market’ it has been shown that success is determined by social influence, i.e. people showed the tendency to prefer music that they perceived was also preferred by many other listeners [15]. Music preferences are also shaped by nationality, language, and geographic location [17]. Interestingly, a geographic flow of music has been detected between cities, where some of them consistently act as early adopters of new music [17]. Over the last fifty years popular music experienced growing homogenization over time with respect to timbre [18], which is *the* fingerprint of musical instruments and was found to exhibit similar statistical properties as speech [19]. Another important application of a quantitative evaluation of trends in the music industry is the development of music recommendation systems that are based on the similarity of artists [20], or on collective listening habits of users of online music databases [21]–[24].

Here we assume that *instrumentational complexity* of a style is related to the set of specialized skills that are typically required of musicians to play that style. Instrumentational complexity of a style increases with (i) the number of skills required for the style and (ii) the degree of specialization of these skills. A highly complex music style, in terms of instrumentation, requires a diverse set of skills that are only relevant for a small number of other styles. A style of low instrumentational complexity requires only a small set of generic and ubiquitous skills, that can be found in a large number of other styles. If a music style requires a highly diverse set of skills, this will to some degree also be reflected in a higher number of different instruments and production technologies. In general, demand for variety translates into a larger number of instruments used in the production process. Desire for uniformity favors a limited variability in instrumentation in a production. Music styles with high instrumentational complexity therefore have large instrumentational variety and at the same time low instrumentational uniformity. It follows that the desire for variety and uniformity are not only relevant for the perception of musical patterns. The notions of variety and uniformity also apply to the instrumentations that musicians use for their pieces. Note that instrumentational complexity can be regarded as a timbral complexity measure and is not informative about, for example, rhythmic, tonal, melodic, or acoustic complexity [12].

In this work we quantify the variety and uniformity of music styles in terms of instrumentation that is typically used for their production. We employ a user-generated music taxonomy where albums are classified as belonging to one of fifteen different music genres that contain 374 different music styles as subcategories. Styles belonging to the same music genre are characterized by similar instrumentation, a fact that has already been exploited in the context of automatic genre detection [25]. We construct a similarity network of styles, whose branches are identified as music genres. We characterize the instrumentational complexity of each music style by its instrumentational variety and uniformity and show (i) that there is a remarkable relationship between instrumentational varieties and uniformities of music styles, (ii) that the instrumentational complexity of individual styles may exhibit dramatic changes across the past fifty years, and (iii) that these changes in instrumentational complexity are related to the typical sales numbers of the music style.

## Results

### Music styles and genres are characterized by their use of instruments

We introduce a time-dependent bipartite network connecting music styles to the instruments that are typically used in that style. The dataset is extracted from the online music database *Discogs* and contains music albums and information on which artists are featured in the album, which instruments these artists play, the release date of the album, and the classifications of music genres and styles of the album. For more information see the methods section and S1 Table in S1 File.

**Figure 1:**
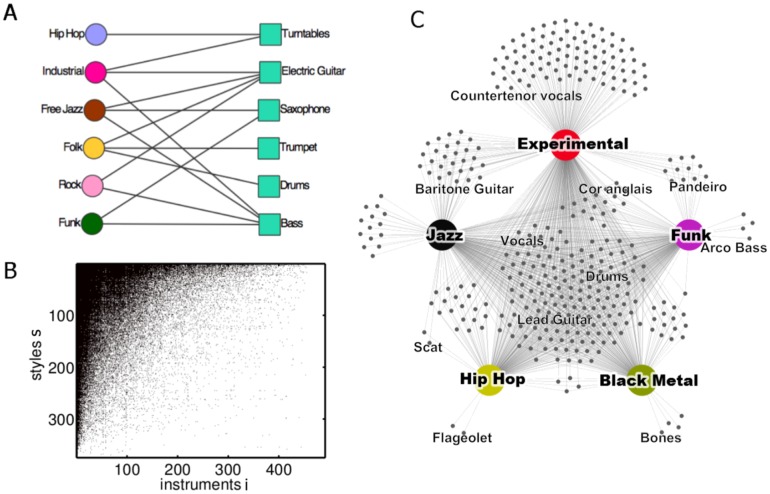
A bipartite network that connects music styles with instruments is constructed. (A) Schematic representation of the data containing the relations between styles and instruments. (B) Visualization of the matrix describing the music production network, 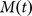. A black (white) field for style 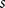 and instrument 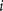 indicates that 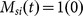. (C) Part of the bipartite network 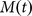 that connects music styles with instruments for a given year 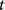. Large nodes represent music styles, small ones instruments. It is apparent that some instruments occur in almost every style while others are used by a substantially smaller number of styles. For instance, there are only two instruments appearing exclusively in ‘hip hop’ among these five styles (for example the flageolet) whereas dozens of instruments are only related to ‘experimental’ (such as countertenor vocals). Vocals, lead guitar, and drums, on the other hand, appear in each of the five styles, whereas bones used as percussion elements only appear in ‘Black Metal’.

We use the following notation. If an album is released in a year from a given time period 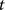, if it is classified as music style 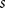, and it contains the instrument 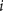, this is captured in the time-dependent *music production network* 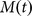, by setting the corresponding matrix element to one, 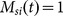. If instrument 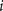 does not occur in any of the albums assigned to style 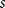 released in time 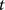, the matrix element is zero, 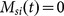. Fig. 1A shows a schematic representation of the relations between several instruments and styles, and Fig. 1B shows the music production network 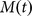 for the years 2004–2010. Let 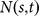 be the number of albums of style 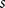 released at time 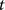. We only include styles with at least 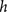 albums released within a given time window, 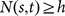. If not indicated otherwise, we choose 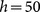. The music production network 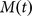 can be visualized as a dynamic bipartite network connecting music styles with instruments. Fig. 1C shows a snapshot of this bipartite network for five different music styles and their instruments. Vocals, lead guitar, and drums appear in each of these styles, whereas for example bones used as percussion element only appear in ‘Black Metal’.

**Figure 2:**
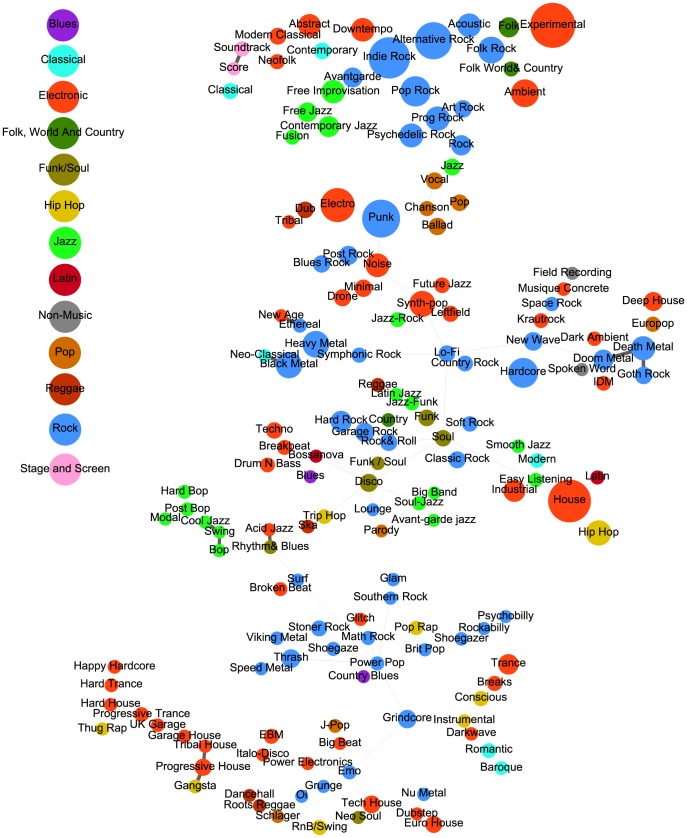
Maximum spanning tree for the style-similarity network, 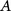, for the years 2004–2010. Nodes represent styles, colors correspond to the genre to which the style belongs, the node size is proportional to the number of albums released for each style, the link strength between two music styles 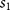 and 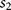 is proportional to 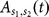. Several clusters are visible. They are identified as styles belonging to ‘rock’, ‘jazz’, or ‘electronic music’ genres.

The similarity of two styles 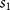 and 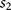 can be computed by the overlap in instruments which characterize both styles at time 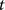, as measured by the similarity network 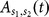 that is defined in Methods. 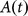 is related to the conditional probability that an instrument that is relevant for one style is also associated to the other style. Fig. 2 shows the maximum spanning tree (MST) of the style similarity network 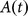 computed for the last time period in the data, *t* = 2004–2010. The size of nodes (styles) is proportional to the number of albums 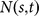 of style 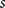 released in that period. The data categorizes styles into genres, see Methods and supporting information S1 Fig. in S1 File. Node colors indicate music genres, the strength of links is proportional to the value of 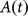. The MST shows several groups of closely related styles that belong to the same genres, such as ‘rock’, ‘electronic music’, or ‘jazz’. Let 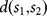 be the shortest path length between two styles 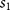 and 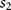 in the MST. The average value of 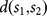 between two styles that belong to the same genre is significantly smaller than the average 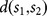 for two styles that do not belong to the same genre. To show this we consider two groups of values of 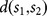 given by whether 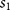 and 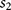 belong or do not belong to the same genre. A *t*-test rejects the null hypothesis that the values in both groups are sampled from distributions with equal means up to a *p*-value of 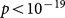, with a smaller average 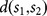 for styles of the same genre. The identified clusters of similar styles can be related to characteristic sets of instruments that define these genres. ‘Jazz’ is mostly influenced by music instruments such as saxophone, trumpet and drums. ‘Rock’ typically involves electric guitars, synthesizer, drums and keyboards, whereas ‘electronic music’ is characterized by synthesizer, turntables, samplers, drum programming, and computers.

### From instrumentational variety and uniformity to complexity

The *instrumentational variety* 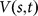 of style 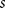 at time 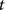 is the number of instruments appearing in those albums that are assigned to 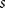, 
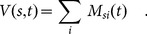
(1)



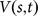
 depends on how many different skills or capabilities of musicians (such as playing an instrument) are typically found within a music style. *Instrumentational uniformity* 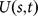 of style 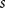 at time 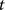 is the average number of styles that are related to an instrument that is linked to style 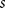, or explicitly 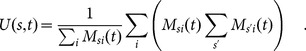
(2)


To put it differently, the instrumentational uniformity of a given style 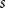 is the average number of styles in which an instrument linked to 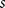 is typically used. Low (high) values of 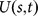 indicate that the instruments characterizing style 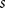 tend to be used in a small (large) number of other styles.


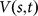
 and 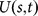 measure different aspects of *instrumentational complexity*. The instrumentational variety 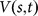 is the degree of style 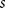 in the bipartite network 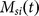 shown in Fig. 1A. 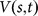 is therefore a *local* network property of a single node. Instrumentational uniformity 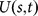 can be interpreted as the average degree of all nodes that represent instruments that are linked to style 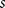 in 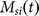. 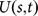 is a *global* network property that can only be computed with the knowledge of the entire network. The indicators 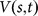 and 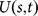 are reminiscent of measures proposed to quantify the complexity of economies of countries by the analysis of bipartite networks that connect countries to their exports of goods [26]. It was shown that changes in indicators resembling 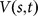 and 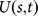 are predictive for changes of national income.

As a measure for the number of sales of an album we use its Amazon ‘SalesRank’, see Methods. The average sales of a given music style 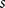, 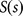, is given by the average SalesRank of albums assigned to style 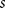, 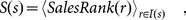
(3)where 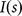 is the index set of all albums 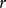 that are assigned to style 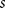. 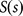 is the average SalesRank of these albums.

*Instrumentational complexity* of a music style can be expressed as the property of having high variety and low uniformity in terms of instrumentation, i.e. the music is produced with a large number of different instruments which only appear in a small number of other styles. Such production processes require musicians with a diverse and highly specialized set of skills. As a complexity index 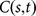 of a style 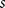 at time 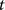 we use 
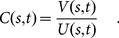
(4)


**Figure 3:**
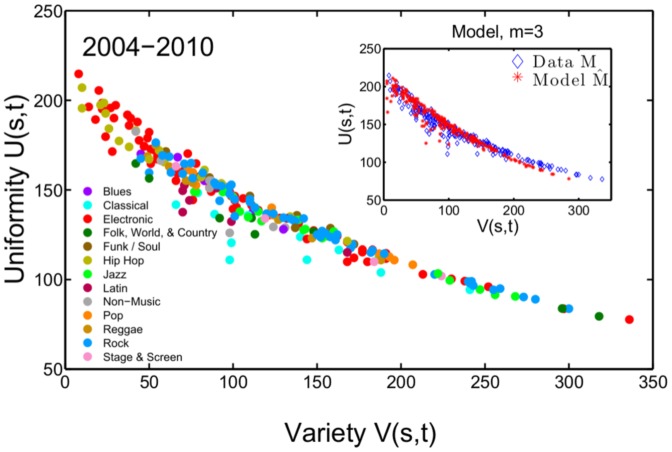
Instrumentational variety 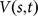 and uniformity 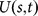 for music styles within the time period *t* = 2004–2010. Music styles collapse onto a line where 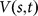 and 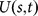 are inversely related. ‘Experimental’ is the music style with the highest instrumentational variety, styles with the lowest levels of variety and highest uniformity values belong to the ‘electronic’ and ‘hip hop’ genres. *Inset:* The values for 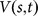 and 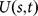 are similar to results from the model.

Fig. 3 shows each style (containing at least 50 albums) at time 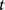 in the 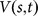-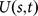 plane for the time window 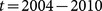. The styles follow a particular regularity: the higher the instrumentational variety 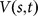 of a music style, the lower its instrumentational uniformity 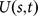. The style with the highest variety is ‘experimental’, a style that categorizes music that goes beyond the frontiers of well established stylistic expressions. Most of the 20 styles with highest variety (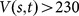) belong to the ‘rock’ genre. Styles with low variety (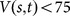) mostly belong to the ‘electronic’ and ‘hip hop’ genres. Interestingly, styles that deviate most from the curved line in Fig. 3 by having a comparably low uniformity correspond to styles such as ‘Medieval’, ‘Renaissance’, ‘Baroque’, ‘Religious’, and ‘Celtic’. These styles are played using unique instruments that require musicians with special training. In Fig. 3 the styles with high complexity can be found in the lower right quadrant of the plot, whereas styles with low complexity populate the upper left quadrant. The negative relation between the local network property 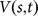 and the global property 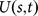 hints at a specific global organization of the music production network 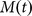. In particular this relation suggests that those music styles with low instrumentational variety 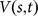 are characterized by instruments that are typically related to a large number of other styles. This finding is consistent with the ’triangular arrangement’ of non-zero matrix elements of 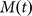 that is apparent in Fig. 1B, where styles and instruments are ordered by their degrees in 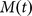, respectively. The non-zero elements in 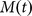 are not evenly spread out over instruments and styles, but instead styles with low degree (low instrumentational variety) are typically related to instruments with a high degree.

### Complexity-lifecycles of music styles

**Figure 4:**
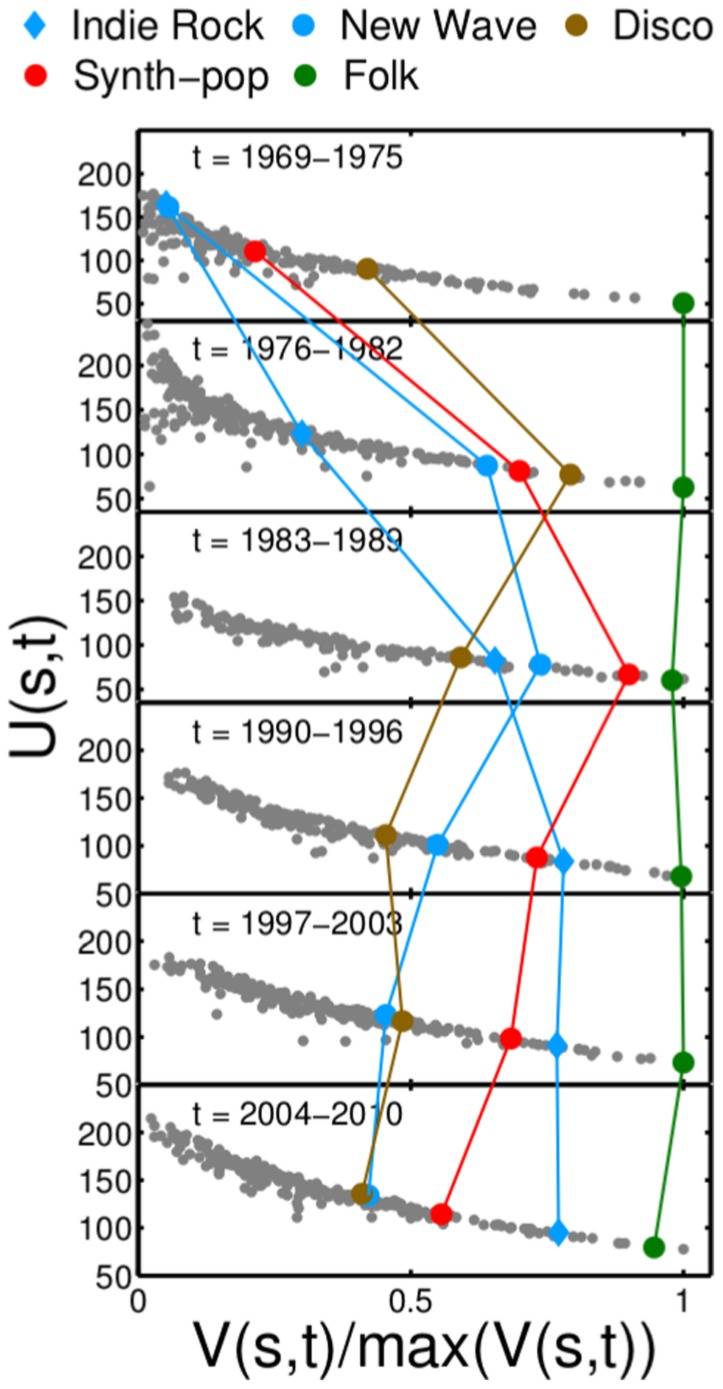
The arrangement of styles in the *V*-*U* plane remains robust over more than fifty years of music history. However, the position of individual styles can change dramatically over time, as it is shown for ‘indie rock’, ‘new wave’, ‘disco’ and ‘synth-pop’. Some styles, such as ‘folk’, show almost no change in their position.

The relationship between instrumental variety and uniformity of styles is remarkably stable over time. Variety 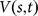 and uniformity 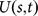 have been computed for six time-windows of seven years, starting with *t* = 1969–1975. For each time period 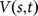 and 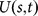 show a negative relation in Fig. 4. Values of 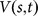 are normalized by 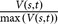 to make them comparable across time. Although this relation is stable over time, the position of individual styles within the plane can change dramatically, as can be seen in the highlighted trajectories of several styles. The evolution of music styles is also shown in the S2 Fig. in S1 File where the trajectory of 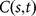 is shown for each style that ranks among the top 20 high instrumentational complexity styles. For example, the style ‘new wave’ sharply increased in complexity rapidly and was popular from the mid-70's to the mid-80's, after which it decreased again. Similar patterns of rise and fall in complexity are found for ‘disco’ and ‘synth-pop’ music. ‘Indie rock’ gained complexity steadily from the 60s to the 80s and remained on high complexity levels ever since. Styles losing instrumentational complexity over time include ‘soul’, ‘funk’, ‘classic rock’, and ‘jazz-funk’. However, other styles such as ‘folk’, ‘folk rock’, ‘folk world’, or ‘country music’ remain practically at the same level of complexity.

**Figure 5:**
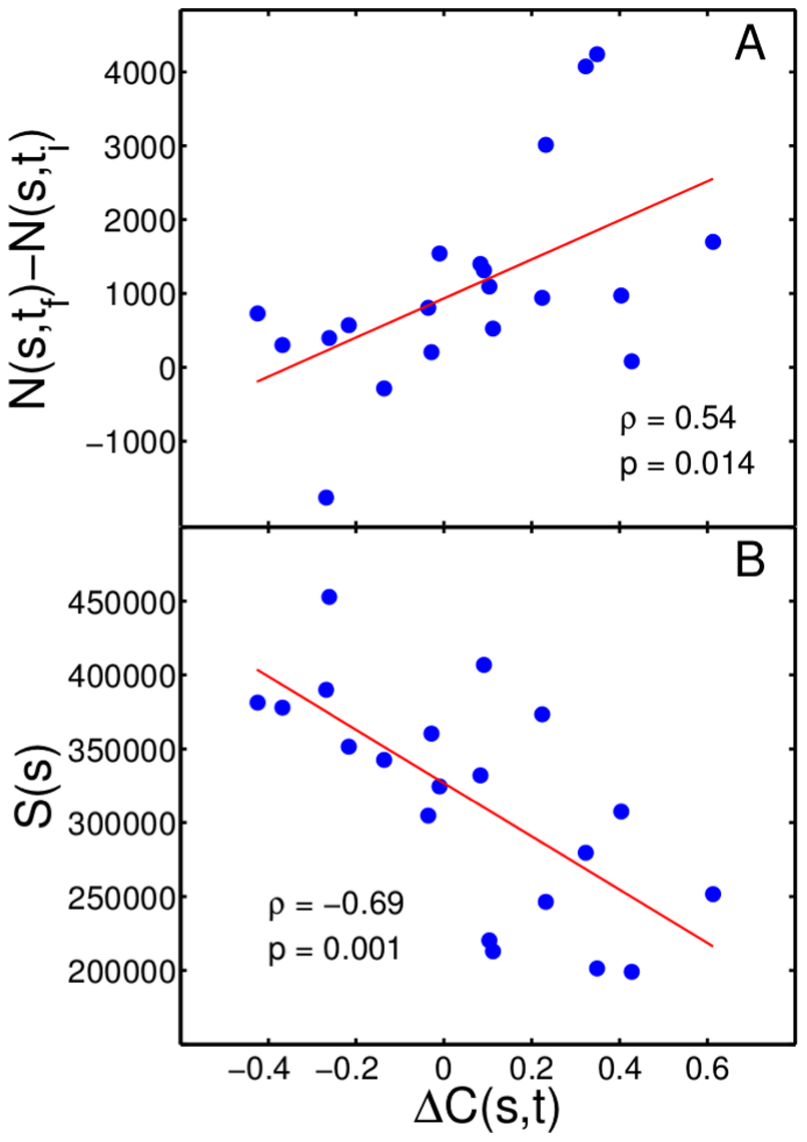
Changes in instrumentational complexity of a style are related to its number of sales and to the number of artists contributing to that style. (A) Changes in number of albums 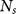 versus changes in instrumentational complexity of music styles 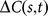. We find a positive trend between 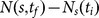 and 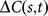 with correlation coefficient 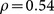 and p-value 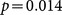. (B) Sales 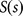 show a negative trend when compared to the change of complexity of music styles with correlation coefficient 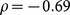 and p-value 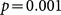.

To understand the mechanisms leading to an increase or decrease in instrumentational variety and uniformity we compute the change in the number of albums for each style between two seven-year windows, 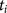 = 1997–2003 and 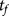 = 2004–2010. The change in number of albums is compared with changes in instrumentational complexity 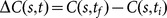, see Fig. 5A. We find that increasing complexity is typically related to an increasing number of albums within that time-span with a correlation coefficient 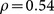 and p-value 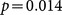. This suggests that styles with increasing complexity attract an increasing number of artists that release albums within that style.

There exists a remarkable relation between changes in instrumentational complexity of a style and its average number of sales. Fig. 5B shows that 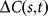 has a negative trend when compared to the average number of sales 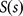 as defined in Equation (3), with correlation coefficient 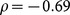 and p-value, 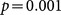. This negative trend is also significant if we define the average number of sales by using the geometric mean in Equation (3), i.e. by taking the average of the logarithmic numbers of sales (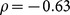, 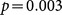). Naively, one could assume that styles with increasing sales numbers show increasing numbers of albums, since they offer more prospect for generating economic revenue. However, the opposite is true, 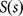 and the change in albums 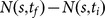 have a significantly negative correlation coefficient, 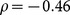 and p-value 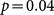. S3 Fig. in S1 File shows a version of Fig. 5 where each data point is labeled by its style. Note that here we take into account only styles 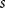 that have at least 1,500 albums in periods 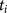 and 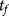 since only for those the average SalesRank can be estimated reliably. Note that there are no significant correlations between the number of albums per style 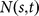 and the indicators 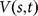 (correlation coefficient 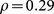 and p-value 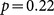), 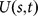 (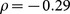, 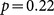), 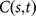 (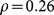, 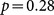), and 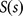 (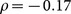, 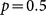). It can therefore be ruled out that the results for changes in the complexity of styles over time, see Fig. 4, and their relation to the average number of sales, shown in Fig. 5A and B, are driven by changes in the number of albums for each style. It is also ruled out that the negative correlation between instrumentational variety and uniformity shown in Fig. 3 (and in S4 Fig. in S1 File for 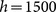) can be explained by a confounding correlation with the number of albums per style.

### A simple model

High instrumentational complexity typically requires musicians with a diverse and highly specialized set of skills. We now show that the results for instrumentational variety and uniformity can be understood with a simple model that explicitly takes the capabilities of artists into account. Therefore we introduce two bipartite networks that can be extracted from the data: the style-artist network 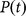 and the artist-instrument network 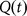. Entries in 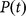 and 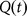 are zero by default. If a given artist 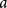 is listed in the credits of an album released at time 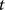 and assigned to style 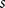, we set 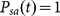. If the artist 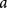 plays instrument 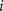 on an album released at 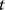, we set 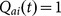. In the model we assume that instrument 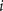 is associated to style 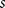 if there are at least 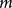 artists which are both related to instrument 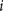 and to style 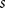. The model music production network 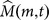 is given by 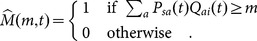
(5)


The parameter 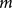 allows to investigate whether the results are driven by spurious connections in 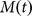, that is by style-instrument relations that are constituted by a very small number of artists. If such spurious connections matter we would expect to find different results for the relations between instrumentational uniformity and diversity, and between instrumentational complexity and number of sales 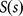 for large values of 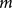. From 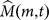 we compute the model variety 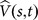 and model uniformity 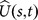. The optimal choice of the threshold 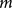 is found by maximizing the goodness-of-fit between data and model, see Methods. The model explains the data best if one assumes that an instrument 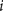 can be associated with a style 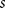, given that there are at least 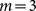 artists that are both related to style 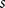 and instrument 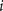. The results for model variety and uniformity for 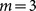 are shown in the inset of Fig. 3. For the bulk of styles, data and model are practically indistinguishable. S4 Fig. in S1 File shows a comparison of data and model for various choices of 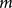 and 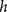. By increasing 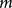 the model instrumentational variety 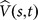 and uniformity 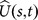 typically decrease. There is a negative trend between 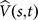 and 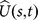 that tends to become steeper with higher values of 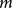.

Note that the results shown in Figs. 3 and 5 can *not* be explained by trivial features of the data such as numbers of instruments or artists per style alone. This is shown by introducing randomized versions of 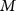 and 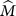. A randomization of 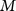 is obtained by replacing each row in 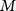 by a random permutation of its elements, we call it 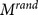. The varieties of each style are the same for 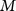 and 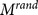, but uniformities will change. Results for the relationship between instrumentational variety and uniformity when computed from 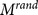 are shown in S5 Fig. in S1 File for two different choices of the threshold 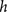 (50 and 1,500, respectively). S5 Fig. in S1 File shows that in these cases there is no inverse relation between instrumentational variety and uniformity for either choice of 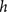, and that the data can not be reproduced. The non-trivial relation between 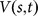 and 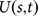 in Fig. 3 is therefore driven by the differing uniformities of music styles and can *not* be explained by variety alone.

A randomized version of the model music production network 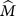, 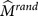, is obtained by replacing both the style-artist network 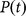 and the artist-instrument network 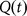 by randomizations. In these randomizations 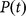 (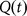) is replaced by a random matrix that has the same size and number of zeros and ones as 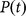 (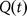), and where each entry is nonzero with equal probability. That is, each artist is assigned a randomly chosen set of instruments and styles while the total number of associations is fixed. S6 Fig. in S1 File shows that in this case we recover an inverse relation between instrumentational variety and uniformity, but the high overlap between data and model disappears under this randomization. There is also no significant correlation between sales 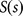 and the change in complexity for the randomized music production network 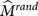. The relationships between instrumentational variety, uniformity, and sales numbers for the various music styles can only be explained by taking the skills of musicians into account, i.e. *who* is able to play *which* instrument under *which* stylistic requirements.

## Discussion

We quantified instrumentational variety and uniformity of music styles over time in terms of the instruments that are typically involved in crafting popular music. We construct a bipartite network that connects styles to the instruments they are typically associated with, the so-called music production network 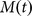. Instrumentational variety is a local network property of 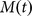, given by the degree of a style in this bipartite network. Instrumentational uniformity of a style is a global network property of 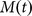 that is related to the average number of styles in which instruments that are connected to this style appear. From the music production network we construct a style-similarity network where styles are linked if they are associated with similar sets of instruments. Clusters of styles in this network correspond to music genres such as ‘rock’ or ‘electronic music’. Instrumentational complexity of a music style is the property of having both, high instrumentational variety, and low instrumentational uniformity. We found a negative correlation between variety and uniformity of music styles that was remarkably stable over the last fifty years. This finding reveals an intriguing relation between local and global properties of the music production network. Styles with low instrumentational variety are characterized by instruments that are typically associated with a large number of other styles. While the overall distribution of instrumentational complexity over music styles is robust, the complexity of *individual* styles showed dramatic changes during that period. Some styles like ‘new wave’ or ‘disco’ quickly climbed towards higher complexity and shortly afterwards fell back, other styles like ‘folk rock’ stayed highly complex over the entire time period. We finally showed that these changes in the instrumentational complexity of a style are typically linked to the sales numbers of the style and to how many artists the style attracts. As a style increases its number of albums, i.e. attracts a growing number of artists, its variety also increases. At the same time the style's uniformity becomes smaller, i.e. a unique stylistic and complex expression pattern emerges. Album sales numbers of a style, however, typically increase with *decreasing* complexity, see Fig. 5B. This can be interpreted as music becoming increasingly formulaic in terms of instrumentation under increasing sales numbers due to a tendency to popularize music styles with low variety and musicians with similar skills. Only a small number of styles in popular music manage to sustain a high level of instrumentational complexity over an extended period of time.

## Materials and Methods

### Data

The *Discogs* database is one of the largest online user-built music database specialized on music albums or discographies. Users can upload information about music albums. A group of moderators assures correctness of the information. Discogs is an open source database and publicly accessible via API or XML dump file released every month. We use the dump file of November 2011 containing more than 500,000 artist and more than 500,000 albums assigned to 374 styles. The data spans more than fifty years of music history, from 1955–2011. Discogs uses a music taxonomy based on two levels, music genres and styles. There are fifteen different genres, such as ‘rock’, ‘blues’, or ‘Latin’. On the second level genres are divided into styles, for instance ‘rock’ has 57 styles including ‘art rock’, ‘classic rock‘, ‘grunge’, etc. ‘Latin’ contains 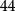 different music styles such as ‘cumbia’, ‘cubano’, ‘danzon’, etc. S1 Fig. in S1 File shows the histogram of the distribution of music styles per genres. For each music album we extract information on the instruments played by artists, the release date of the record, and the music genres and styles assigned to the album. The data is grouped into time windows of seven years, e.g. the last time-step contains data on albums released between 2004–2010, and so on. S1 Table in S1 File provides some descriptive statistics of the dataset.

To measure the average sales numbers of music styles we use a dataset that contains information on the Amazon SalesRank of music albums as of 2006 [27]. The Amazon SalesRank can be thought of as a ranking of all records by the time-span since an item last sold [28]. Albums in the Discogs dataset are assigned their Amazon SalesRank by matching album titles between the two datasets. As the Amazon SalesRank dataset only contains information on album titles, it was matched to entries in the Discogs dataset by choosing only albums whose title appears only once in both datasets.

### Style similarity network

The *style similarity network* 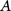 quantifies how similar two music styles are in terms of their instrumentation. A weighted link in matrix 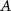 connects two music styles, 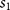 and 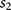, and is defined as the number of instruments they have in common, divided by the maximum value of their respective varieties 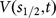. At a given time 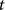, the entries in 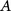 are given by 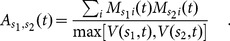
(6)


The value of 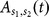 is the minimum of the two conditional probabilities that an instrument related to style 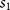 (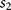) is also related to style 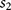. The smaller of the two conditional probabilities is used to avoid spurious results from styles with low instrumentational variety. To visualize the network of music styles we compute the maximum spanning tree (MST) for 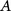. This visualization strategy is similar to the one presented in [29].

### Goodness-of-fit between data and model

We use 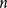 data bins 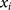 for the instrumentational variety with intervals of size one, 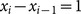. We define the binned uniformity 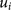 (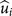) for the data (model) as the average uniformity of all styles 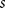 with variety 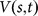 (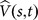) 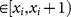. The average squared residuals 
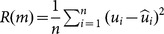
 are then calculated for 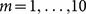. For 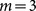 the value of 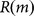 assumes its minimum, see S4 Fig. in S1 File.

## Supporting Information

S1 File **Supporting information.**
(PDF)
