# Supplementary material for: Instrumentational Complexity of Music Genres and Why Simplicity Sells
Source: PLoS One. 2014 Dec 31;9(12):e115255. doi: 10.1371/journal.pone.0115255 (PMC4281146; doi:10.1371/journal.pone.0115255)
Supplement: S1 File — Supporting information. (PDF) [file pone.0115255.s001.pdf]

Supporting Information

I. DATA

Discogs.com is a comprehensive, user-built music database with the aim to provide cross-referenced discographies of all labels and artists. As of April 2014, more than 189,000 people have contributed to this collection. We work with an XML dump of the database from November 2011. The number of data entries is shown in Tab. S1. The dataset includes more than half a million artists and albums spanning the years 1955-2011, as well as almost 500 instruments.

TABLE S1: Overview of data extracted from *Discogs.com*

Data type	Number of entries
Artists	580060
Albums	536422
Instruments	491
Styles	374
Genres	15
Years	1955-2011

Discogs uses a music taxonomy for albums based on two levels. On the first, highest level in the taxonomy there are 15 different music genres, for instance 'rock', 'blues', or 'electronic'. On the second level the genres are broken down into 374 different styles, for example 'drum and bass' is a style of the genre 'electronic'. Figure S1 shows a rank-frequency plot of the genres and the number of styles they contain. The genres 'electronic' and 'rock' have the largest number of styles (more than fifty), whereas 'brass & military', 'stage & screen', and 'children's music' have the smallest number of styles. The genre and style information for albums is also entered by users, who may choose from a pre-specified list of styles. This list of styles is generated by discogs users as the outcome of a collaborative and moderated process.

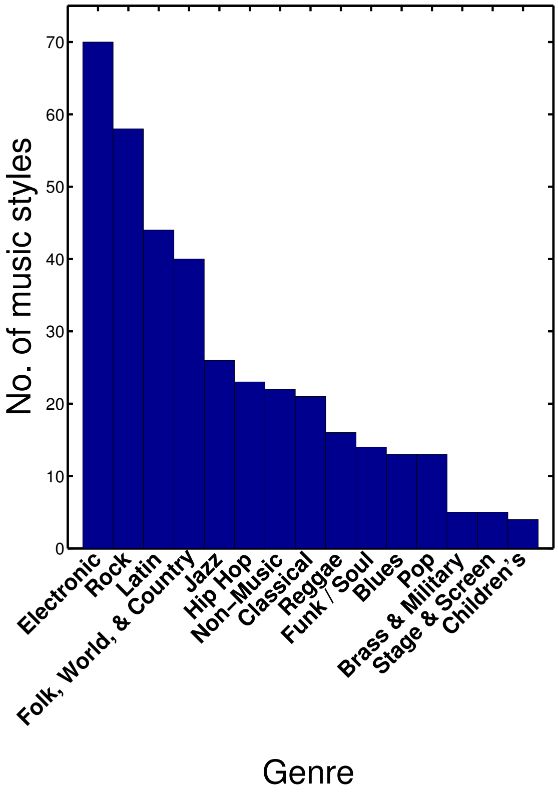

FIG. S1: Rank-frequency plot of the number of styles per genre. 'Electronic' and 'rock' genres contain the largest numbers of styles, 'children's music' the least number of styles.

II. COMPLEXITY LIFE-CYCLES OF MUSIC STYLES

Figure S2 shows the trajectories for instrumentational complexity $C(s,t)$ for each style which was ranked at least once among the twenty styles with highest complexity. 'Experimental', 'folk', and 'folk rock' rank among the top-5-variety-styles in each time window. 'New wave' and 'indie rock' start at a variety rank around 200 in 1969-1975 and show a stark increase in complexity over the next time-windows. In 1983-1989 'new wave' reaches rank 5, 'indie rock' is ranked #15 in this time window. However, afterwards their trajectories diverge drastically. 'New wave' goes quickly down in complexity until it reaches a rank of 73 in 2004-2010, whereas 'indie rock' continues to climb up to rank 9 in the last time window. The styles 'disco' and to a lesser extent 'synth-pop' show the same pattern of variety changes as 'new wave', i.e. a rapid increase followed by an equally rapid decrease. 'Alternative rock' and 'downtempo' show complexity changes similar to 'indie rock', namely continual increases. Other styles show a decline in complexity over time. For example 'soul',

‘classic rock’ and ‘funk’ have complexity ranks in the range 10-20 in 1969-1975, while none of them is ranked in the top 50 in 2004-2010.

Figure S3 shows a version of Figure 5 from the main text with labels of the music styles for data points. It becomes apparent that the styles ‘euro house’, ‘synth-pop’, ‘disco’, ‘pop rock’, and ‘hard rock’ exhibit decreasing complexity, increased average sales numbers $S(s)$, and decreased numbers of albums. ‘Experimental’, ‘ambient’, ‘alternative rock’, and ‘hip hop’ show the largest increases in complexity over time, while their averages sales decrease and the number of related albums increases.

Results for the distribution of styles in the instrumentational variety-uniformity plane are compared for data and model of Equation 5 in Figure S4. In the left column of Figure S4 results for a threshold value of $h = 50$ are shown, the right column shows results for $h = 1500$. Each row corresponds to a different value of $m = 1, 2, 3, 5, 10$. It is apparent that for both thresholds h the highest overlap between data and model is found for $m = 3$. A model where it takes at least three musicians playing a given instruments and releasing albums in a given style, in order to constitute a style-instrument-relation, describes the data best.

III. RANDOMIZATION RESULTS

The distribution of variety and uniformity values of music styles for the music production network M obtained from the data is compared to the distribution of styles from the randomized production network M^{rand} in Figure S5 for two threshold values, $h = 50$ and $h = 1500$. The randomization destroys the negative correlation between variety and uniformity. The styles have similar levels of uniformity, independent from their variety values, and the results for M^{rand} resemble the results obtained from M only very poorly.

Results for the distribution of variety and uniformity of music styles for data and for the randomized model production network $\widehat{M}^{rand}$ are shown in Figure S6 for two threshold vales, $h = 50$ and $h = 1500$. The randomization $\widehat{M}^{rand}$ also shows a negative correlation between $V(s, t)$ and $U(s, t)$, but especially the uniformity values are strongly underestimated in the randomized model when compared to the data. The correlation between complexity change and sales numbers is destroyed by the randomization in $\widehat{M}^{rand}$, as is shown in the bottom row in Figure S6.

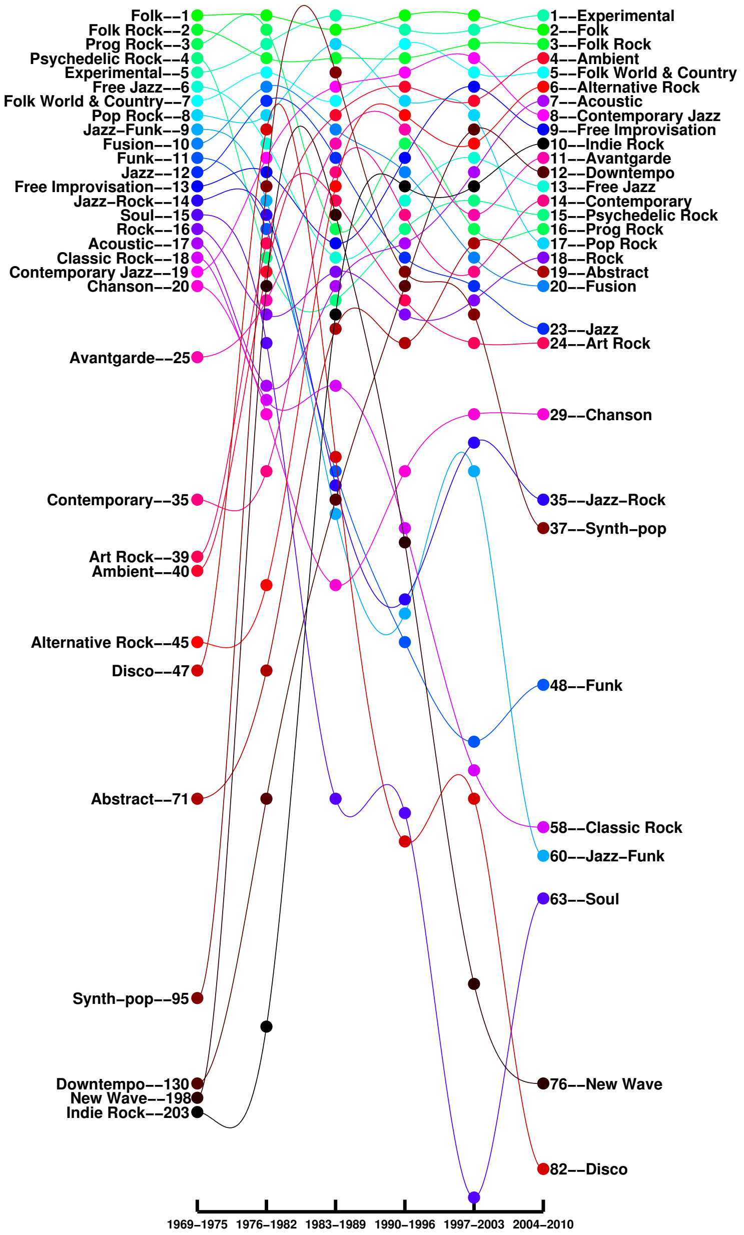

FIG. S2: Music styles are ranked according to their instrumentational complexity over thee last fifty years. Styles are ranked according to their complexity in each of the studied time windows, and the changes in complexity are shown as trajectories for each style that ranked at least once among the top 20 in terms of complexity. ‘Experimental music’, ‘folk’ and ‘country’ are nearly stationary, while ‘indie rock’, ‘new wave’, or ‘disco’ changed their complexity-ranks dramatically.

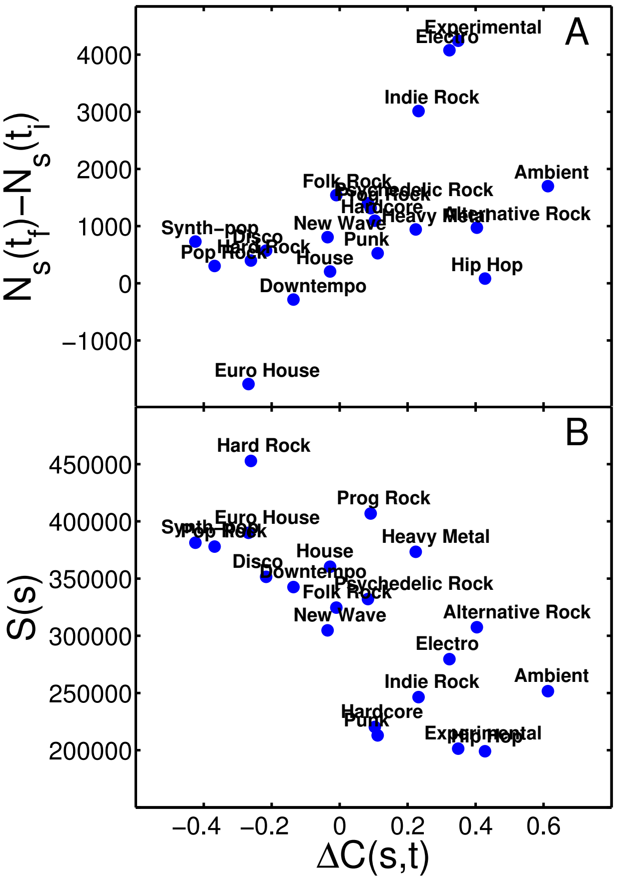

FIG. S3: Changes in instrumentational complexity of a style are related to its number of sales and to the number of artists contributing to that style. This figure shows the same as Figure 5 in the main text with labels for the music styles.

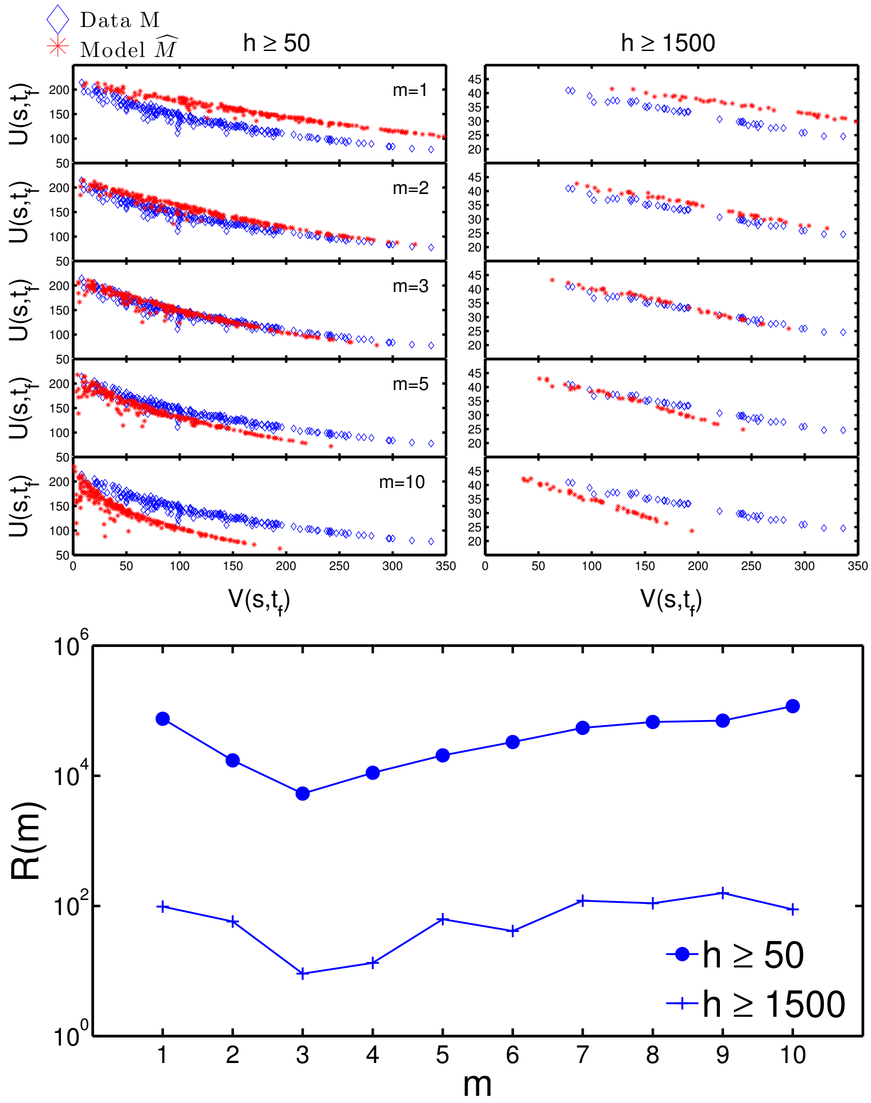

FIG. S4: Variety and uniformity values for data and model for threshold values of $h = 50$ (left) and $h = 1500$ (right). Each row corresponds to a different value of $m = 1, 2, 3, 5, 10$. The average squared residuals $R(m)$ are calculate for $m = 1, ..., 10$ showing that for $m = 3$ the model describes the data independently from the threshold h, meaning it is enough m=3 artist to associate and instrument i with a style s.

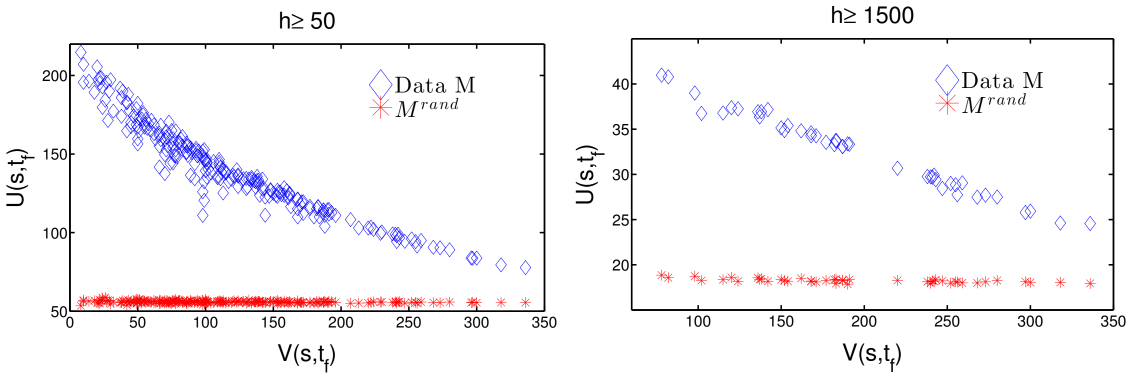

FIG. S5: The distribution of styles in the $V-U$-plane computed from the data M and its randomization M^{rand} shows that the negative correlation between V and U is destroyed by randomizing the instruments associated to each style. The relation between V and U is therefore the result of a nontrivial structure captured by the uniformities of styles.

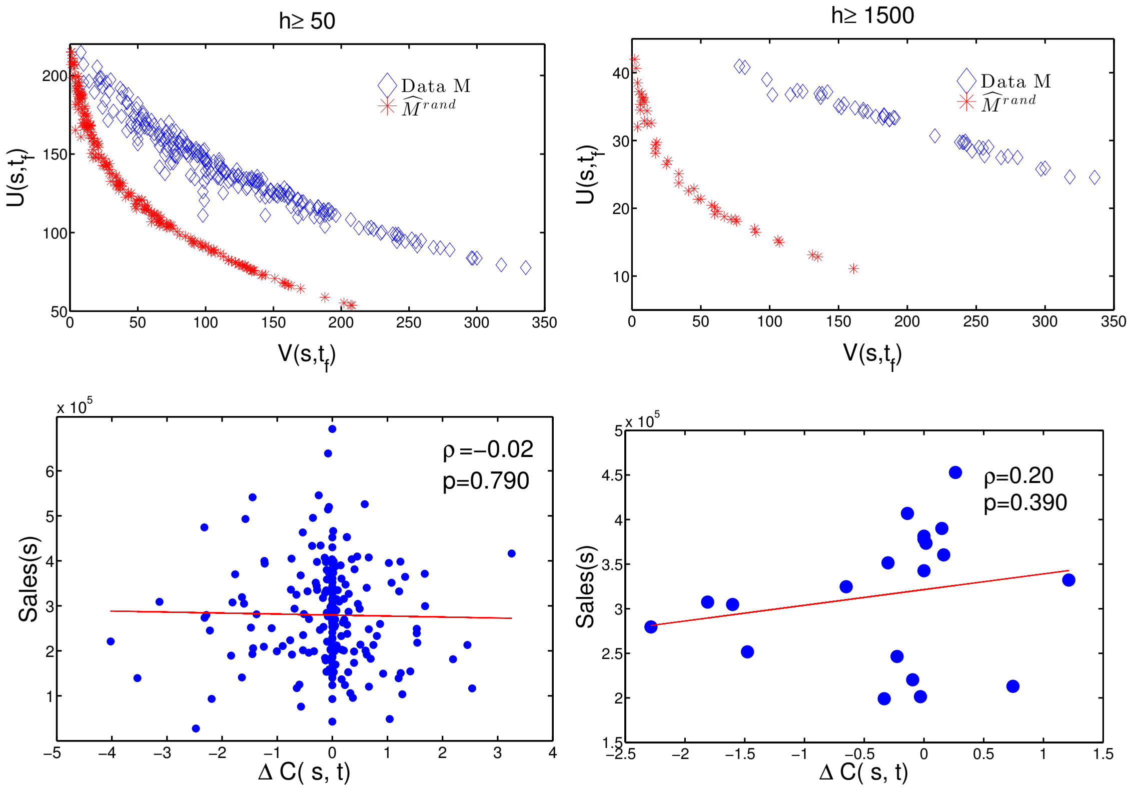

FIG. S6: Comparison between data and the randomized model $\widehat{M}^{rand}$. *Top row:* The distribution of styles in the V-U-plane computed from the randomized model $\widehat{M}^{rand}$ shows that the negative correlation between V and U is preserved for both threshold vales, $h = 50$ (left) and $h = 1500$ (right). However, in particular the uniformity values of styles are much smaller under randomization, when compared to the data. *Bottom row:* There is no correlation between complexity change and change in sales numbers for both thresholds, $h = 50$ and $h = 1500$.
